# Editorial: Metallic Micronutrient Homeostasis in Plants

**DOI:** 10.3389/fpls.2019.00927

**Published:** 2019-07-17

**Authors:** Hannetz Roschzttardtz, Manuel González-Guerrero, Diego F. Gomez-Casati

**Affiliations:** ^1^Facultad de Ciencias Biológicas, Pontificia Universidad Católica de Chile, Santiago, Chile; ^2^Centro de Biotecnología y Genómica de Plantas (UPM-INIA), Universidad Politécnica de Madrid, Madrid, Spain; ^3^Centro de Estudios Fotosintéticos y Bioquímicos (CEFOBI-CONICET), Universidad Nacional de Rosario, Rosario, Argentina

**Keywords:** metals, plant nutrition, metal accumulation, frataxin, nodule, medicago

Transition elements, such as copper, iron, or zinc, are essential nutrients for plants. They participate in every biological process. However, intracellular metal levels must be maintained within a narrow physiological concentration. Too little, and not enough cofactors are available to the cell; too much, and Fenton-type reactions and mismetallation events will disrupt many cellular processes. As a result, plants have developed complex systems to control metal uptake and to deliver them to all tissues and cells. In this Research Topic, we have collected some of the most recent work furthering our understanding of Metallic Micronutrient Homeostasis in Plants.

Plant dependence on metals contrasts to the common low bioavailability of these nutrients in many soil types. Growing evidence indicates that plants have optimized the use of metals facilitating their relocation from one cellular compartment to another, depending on their need in different physiological processes. In this Topic, Zhang and Krämer have reanalyzed available bioinformatic data to show that there is a diurnal regulation of the translation of proteins involved in Fe-S metabolism in *Arabidopsis thaliana*, including that of frataxin, a protein involved in iron transfer in Fe-S protein biogenesis, and also in copper control in mitochondria and chloroplasts, as reviewed in this issue by Gomez-Casati et al. Iron is not the only metal micronutrient that is regulated by day-night cycles. Copper homeostasis is also controlled in this way, the likely consequence of plastocyanin synthesis for photosynthesis, and the toxic effect of copper in Fe-S synthesis. Andrés-Colás et al. showed that copper transporter COPT3 transcription is controlled in this way, in a process that is dependent of copper availability to the plant and on transcription factor TCP16.

The study of metal (re)distribution in plants can be greatly assisted by metal-imaging methods. Using X-ray fluorescence approaches, Vigani et al. showed how iron deficiency affects zinc distribution in leaves. With a similar approach, Ibeas et al. have determined the iron distribution in *Chenopodium quinoa* seeds and used it to validate the Perls-DAB histochemical method to visualize iron. This approach not only led to illustrate how iron distribution in seeds changed during dicot evolution but showed how subcellular iron distribution changes during embryo development (Ibeas et al.). Metal imaging approaches are also important to evaluate how metal nanoparticles could enter into trophic chains through plants, and the physiological effects that might cause there (Mosa et al.).

The evolving metal distribution in a cell and an organism could also reflect how some metalloenzymes are expressed with different demand for metal cofactors. For this, we would need high throughput metalloproteomics methods to identify the precise nature of the accepting metalloprotein to put into context many of the observed phenotypes. For instance, symbiotic nitrogen fixation requires one or several zinc-proteins that although dependent on zinc transfer into the endoplasmic reticulum of nodule cells, exert their effect in nitrogen-fixing symbiosomes and in nodule development (León-Mediavilla et al.).

Hormones have been known to control metal homeostasis, but the precise mechanisms of how hormone levels are controlled by metals still remains elusive. In this topic, García et al. reported how long-distance iron trafficking mediated by OPT3 could control ethylene metabolism as well as GSNO levels. In addition, micronutrients such as silicon, modulate hormone levels to overcome abiotic stress such as chilling and also improve the nutrition by metals (particularly zinc and manganese) (Moradtalab et al.).

In summary, the work shown in this Research Topic illustrates our advances in defining how cells and organisms control and redistribute essential metal nutrients. This distribution is affected by biotic and abiotic interactions, hormones and responds to different physiological states of plant development. The work also illustrates the need to have new methods to determine and visualize metals, as well-new developments in metalloproteomics and in metal-speciation analyses.

## Author Contributions

All authors listed have made a substantial, direct and intellectual contribution to the work, and approved it for publication.

### Conflict of Interest Statement

The authors declare that the research was conducted in the absence of any commercial or financial relationships that could be construed as a potential conflict of interest.

